# The unidirectional causality influence of factors on PM_2.5_ in Shenyang city of China

**DOI:** 10.1038/s41598-020-65391-5

**Published:** 2020-05-21

**Authors:** Hongmei Yang, Qin Peng, Jun Zhou, Guojun Song, Xinqi Gong

**Affiliations:** 1 Institute for Mathematical Sciences, Renmin University of China, Beijing, 100872 China; 2 Department of mathematics, Changji University, Xinjiang, 831100 China; 3 Institute of Geographic Sciences And Natural Resources Research,Chinese Academy of Sciences, Beijing, 100101 China; 4 Geographical and environmental school of Tianjin normal university, Tianjin, 300387 China; 5 School of Environment and Natural Resources, Renmin University of China, Beijing, 100872 China; 6 Beijing Advanced Innovation Center for Structural Biology, Tsinghua University, Beijing, 100091 China

**Keywords:** Environmental sciences, Environmental social sciences

## Abstract

Air quality issue such as particulate matter pollution (PM_2.5_ and PM_10_) has become one of the biggest environmental problem in China. As one of the most important industrial base and economic core regions of China, Northeast China is facing serious air pollution problems in recent years, which has a profound impact on the health of local residents and atmospheric environment in some part of East Asia. Therefore, it is urgent to understand temporal-spatial characteristics of particles and analyze the causality factors. The results demonstrated that variation trend of particles was almost similar, the annual, monthly and daily distribution had their own characteristics. Particles decreased gradually from south to north, from west to east. Correlation analysis showed that wind speed was the most important factor affecting particles, and temperature, air pressure and relative humidity were key factors in some seasons. Path analysis showed that there was complex unidirectional causal relationship between particles and individual or combined effects, and NO_2_ and CO were key factors affecting PM_2.5_. The hot and cold areas changed little with the seasons. All the above results suggests that planning the industrial layout, adjusting industrial structure, joint prevention and control were necessary measure to reduce particles concentration.

## Introduction

Air pollution has become one of the severe environmental problems in China. People in China have to cope with high levels of PM_2.5_ (Particulate matter smaller than 2.5 micrometers in diameter). It not only induces the increase of low-visibility days^1–3^, but also penetrates lungs and does harm to respiration, cardiovascular, cerebral vascular and nervous systems^4–7^. Studies show that atmospheric particulate matter is closely related to mortality for lots of causes^8–11^. In addition, Particulate matter possibly has large influence on regional and global climate change^12–14^, ecosystems^15^, economic development^16^ and so on. Thus, Chinese government has established air quality monitoring stations in many cities to monitor pollutants (including PM_2.5_, PM_10_, SO_2_, NO_2_, CO and O_3_) mass concentration, published the relative observed data online from January 2013. These air quality data played an important role on analyzing local air pollution situation and establishing air pollutant mass concentration prediction model^17^.

As previous studies have shown, pollutants sources emission, external transport, meteorological conditions and secondary generation of air pollution were important factors influencing atmospheric particulate matter mass concentration. In particular, meteorological conditions can diffuse, dilute and accumulate air pollutant mass concentration at a large extent. Secondary generation will aggravate air pollutants mass concentration^18,19^. Therefore, the study of the distribution characteristics of air pollutants, the relationship between meteorological conditions and air pollutants mass concentration, as well as the relationship between different air pollutants, can be more helpful for making effective control measures to reduce air pollution. China has the largest population in the world. With the rapid development of economy and the unprecedented urbanization, air pollution in many eastern cities in China was becoming more and more serious. In recent years, the problem of urban air particles in air pollution has become the focus of the problem solved. Every city is trying to explore the temporal and spatial distribution of haze and analyze the causes of its impact. Real-time monitoring data from air quality monitoring station were important for analyzing detailed variation in the scale of cities, including annual, monthly and daily variation^20^. Wang *et al*. analyzed the spatial and temporal changed of PM_2.5_, PM_10_, SO_2_, NO_2_,CO and O_3_ in 31 provincial capitals city of China from 2013 to 2014^21^. Zhang *et al*. explored that spatial distribution of the annual PM_2.5_ concentration in 190 cities of Chinese, PM_2.5_ concentrations were higher in northern than in southern regions, and lower in coastal areas than in inland areas, and it has significant seasonal variations^22^. Zhang *et al*. suggested that spatial and temporal patterns of PM_2.5_ in 190 Chinese cities were positively correlated with its population size and polluting emissions, and negatively correlated with precipitation and wind speed^23^. Yan *et al*. found that temporal and spatial variation of PM_2.5_ in Beijing, and the correlation between PM_2.5_ and meteorological factors in different seasons^24^. Huang *et al*. observed that spatial-temporal change patterns of PM_2.5_ in Beijing from August 2013 to July 2014, and the relationship between meteorological factors with PM_2.5_^25^. Some studies have analyzed the correlation between air pollutants and meteorological factors. Yang *et al*. pointed out that it played a crucial role of interaction between PM_2.5_ and meteorological factors on analyzing air pollution in 68 major cities and 7 geographical regions in China, and meteorological factors included relative humidity, surface pressure, wind speed and temperature, the relationship between meteorological factors with PM_2.5_ has spatial and seasonal variations^26^. Li *et al*. indicated that the relationships between particulate matters with certain meteorological factors in Chengdu city Sichuan province, there was positive correlation between particulate matter and atmospheric pressure, negative correlation between particulate matter and temperature and wind speed, no significant correlation between particulate matter and relative humidity^27^. Chen *et al*. found that PM_2.5_ mass concentration in the Jing-Jin-Ji region has a certain seasonal variation, and meteorological factors have strong correlation with PM_2.5_ mass concentration. Moreover, studies have shown that the higher the PM_2.5_ mass concentration, the greater influence of meteorological factors on PM_2.5_^28^. Similar studies conducted in other cities, such as Beijing, Shanghai, Guangzhou and Wuhan^25,29–31^.

However, a lot of numerical modeling works have also been carried out to quantify the source contributions to the ambient PM_2.5_ in China. Wang *et al*. used PMF model to deduce the contribution sources of PM_2.5_, then they used backward trajectory model to identify the directions of these contribution sources, and determined that the distribution rates of different directions and sources are different^32^. Dou *et al*. applied chemical mass balance receptor model (CMB) to analyze the contribution of PM_2.5_. Source analytical results show that there are many main sources of PM_2.5_ in Xining during the observation period. It mainly includes contribution rate of dust is 26.4%, contribution rate of coal dust is 14.5%, contribution rate of motor vehicle exhaust is 12.8%, contribution rate of secondary sulfate is 9.0%, contribution rate of biomass combustion is 6.6%, the contribution ratio of secondary nitrate is 5.7%, contribution ratio of steel dust is 4.7%, etc. Suggestions put forward to strictly control the pollution sources such as local coal burning and motor vehicles, and control the open source pollution dominated by dust^33^. Chen *et al*. Combines with the chemical mass balance model (CMB) to study the pollution level and main pollution sources of PM_2.5_ in Xingtai city. The results show main sources of PM_2.5_ are soot dust (25%), vehicle exhaust (11%), dust (9%), soil wind dust (3%) and construction dust(2%) during the observation period. The suggestions are that measures should took to control coal burning, dust and industrial production, as well as vehicle emissions^34^. Relevant studies have shown that the distribution of contribution sources of PM_2.5_ is significantly different in different regions^35^.

Up to now, there are still great uncertainties about the temporal and spatial distribution of atmospheric particulate matter, possible influencing factors and quantify the source contributions to PM_2.5_, because different cities have different urban human characteristics and natural environmental factors, there is no unified conclusion on the causes of haze. A large number of literatures have only studied the important factors affecting the particle concentration, which considered as the sum of all explanatory variables and the relationship between them is direct. There are few studies on the indirect effects of particulate concentration. Without considering any indirect effects, important factors can hidden. Therefore, this paper uses path analysis to analyze the direct and indirect effects of a series of explanatory variables, to better analyze the potential variables affecting the particle mass concentration and better describe the complex relationship with particles^36^.

The northeast region is a traditional industrial area in China, accounting for 13% of China’s land area. At the same time, the northeast region is adjacent to countries in Eastern Europe and Asia, so the air pollution problem is more serious, and it is easy to rise to international disputes. Compared with other places, many cities in the northeast region have to face the serious pollution problem in recent years especially Shenyang city. Shenyang city is the largest city in northeast region of China. However, there is very little research on air quality in the northeast. So air pollutants concentration data (PM_2.5_, PM_10_, SO_2_, NO_2_, CO and O_3_) and meteorological parameters collected from 11 air quality monitoring stations within Shenyang city in 2017. Correlation analysis, path analysis and spatial autocorrelation used to analyze influencing factors of particulate matter pollution. The conclusions of this research provided reference for environmental management decisions.

According to the environmental air quality standard (GB 3095-2012) modification list about the requirement in China, particulate matters mass concentration were divided into four levels: good level (PM_2.5_ < 35, PM_10_ < 50),fair level (35 < PM_2.5_ < 75, 50 < PM_10_ < 150), mild level (75 < PM_2.5_ < 115, 150 < PM_10_ < 250), moderate level (PM_2.5_ > 115, PM_10_ > 250).

## Results

### Overview of particulate matter data

Firstly daily average PM_2.5_ and PM_10_ mass concentrations at 11 monitoring stations in Shenyang were summarized. It found that annual mean PM_2.5_ and PM_10_ mass concentrations were 49 μg/m^3^ and 84.9 μg/m^3^, respectively during 2017 at Shenyang. And daily average of PM_2.5_ in summer and winter was 25.7 μg/m^3^ and 63.9 μg/m^3^. The mass concentration in winter was about 2.5 times that in summer.The daily average PM_2.5_ mass concentration ranged from 2.7 μg/m^3^ to 247.8 μg/m^3^, and the lowest concentration was appeared in Donglinglu station, the highest concentration was in Liaoshenxilu station, which were located in eastern region and western region, respectively. The daily average PM_10_ mass concentration varied widely from 2.0 μg/m^3^ to 514.3 μg/m^3^, and the lowest concentration appeared in Donglinglu station and the highest concentration appeared in Jingshenjie station. Jingshenjie station located in the western region. It could be seen that there were great differences in spatial distribution of atmospheric particulate matter in Shenyang. In addition, the median of the atmospheric particulate matter mass concentrations in each monitoring station was far lower than their average value, indicating that the daily average value of the atmospheric particles mass concentrations was a right-skewed distribution. The difference in PM_2.5_ and PM_10_ mass concentrations of each monitoring station had obviously reached the corresponding standard. PM_2.5_ and PM_10_ of Senlinlu station met good level standard (PM_2.5_ < 35 μg/m^3^, PM_10_ < 50 μg/m^3^) with the percentage of 60.7% and 44%, respectively, and significantly higher than other stations reaching good level standard. At the same time, the stations with the lowest proportion of PM_2.5_ and PM_10_ reaching the good level standard were Xinxiujie station, with only 35.7% and 12.5% reaching the standard respectively. For fair level of PM_2.5_ (75 μg/m^3^) and PM_10_ (150 μg/m^3^), just the opposite. Therefore, it indicated better air quality that the highest proportion with two levels(good level and fair level) was Senlinlu station, and worse air quality that the lowest proportion with two levels (good level and fair level) was Liaoshenxilu station, Wenhualu station and Xinxiujie station.

**Figure 1:**
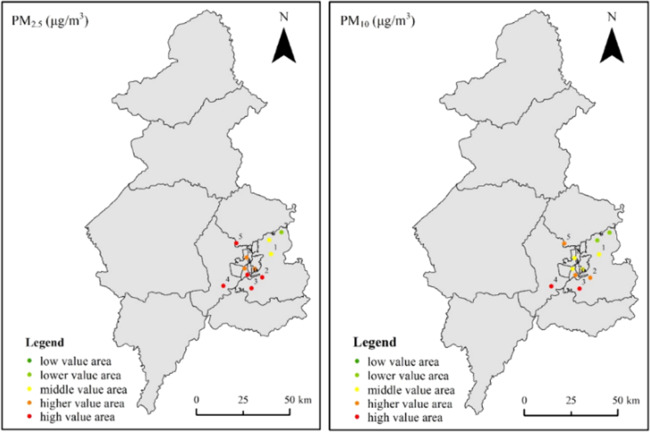
The spatial variation of particulate matter mass concentration.

Figure 1 further showed the spatial variation of PM_2.5_ and PM_10_. The station numbers showed in Table 1. It shown that Senlinlu station and Yunonglu station in northern of Shenyang had the best air quality, and Liaoshenxilu station in western of Shenyang and Wenhualu station in central of Shenyang had the worst air quality. That was, PM_2.5_ and PM_10_ decreased gradually from south to north and from west to east.

**Table 1:** Air quality monitoring stations in different area of Shenyang.

Station number	Station name	Latitude/degree	Longitude/degree	Area
1	Donglinglu	41.50 N	123.35E	Eastern rural area
2	Hunnandonglu	41.44 N	123.30E	Southern urban area
3	Xinxiujie	41.41 N	123.25E	Southern urban area
4	Liaoshenxilu	41.44 N	123.14E	Western urban area
5	Jingshenjie	41.55 N	123.22E	Western rural area
6	Yunonglu	41.54 N	123.35E	Northern rural area
7	Senlinlu	41.56 N	123.41E	Northern rural area
8	Lingdongjie	41.50 N	123.25E	Central urban area
9	Taiyuanjie	41.47 N	123.24E	Central urban area
10	Wenhualu	41.45 N	123.24E	Central urban area
11	Xiaoheyan	41.47 N	123.28E	Central urban area

### The temporal-spatial variation of particulate matter mass concentration

**Figure 2:**
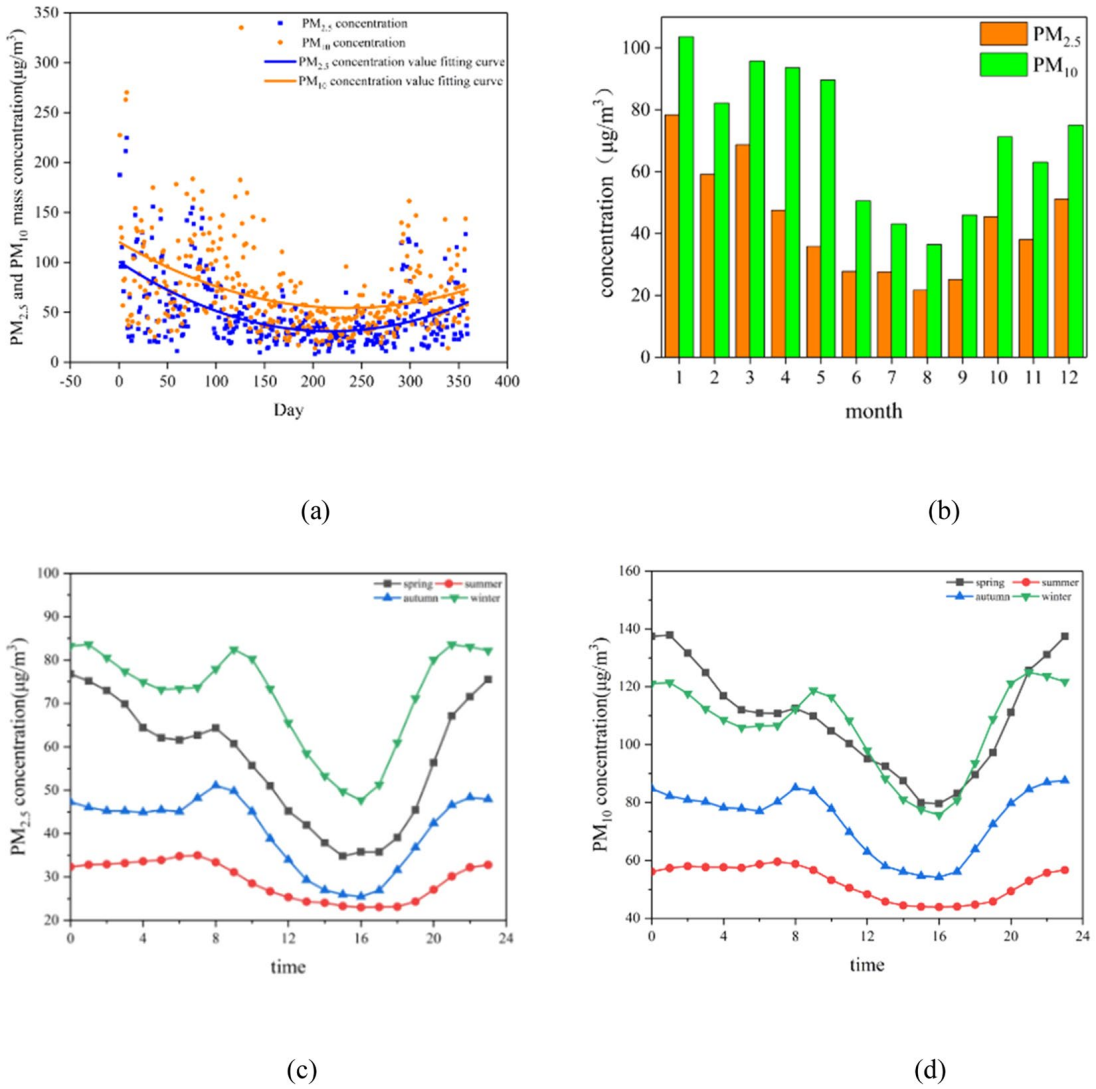
(**a**) Annual variation patterns of daily average particulate matter mass concentrations. (**b**) The variations of monthly average particulate matter mass concentration. (**c**) and (**d**) are the diurnal variations of PM_2.5_ mass concentrations and PM_10_ mass concentration in four seasons.

As shown in Fig. 2(a), the annual temporal variation of PM_2.5_ and PM_10_ presented a saddle-like distribution, with most relatively high value appeared in January, November and December, and most relative low value occurred in July and August. While, the peak value about PM_2.5_ and PM_10_ observed as the section of overview of particulate matter data. Throughout the year, most PM_2.5_ and PM_10_ mass concentrations were at a mild or fair pollution level on most days. The high value in winter mainly due to winter heating and traffic emissions, the low value caused by meteorological factors. The binomial fitting formula was ${\rm{y}}=a{x}^{2}+bx+c$, where y was the dependent variable, x was the independent variable, a, b and c were the coefficients. The fitting results showed that: daily concentration of PM_2.5_ and PM_10_ conformed to the quadratic function, opening up, symmetry axis of the fitting function was concentrated in July and August.

Figure 2(b) showed monthly average variation patterns of PM_2.5_ and PM_10_. PM_2.5_ and PM_10_ presented a similar variation trend, and both of them with relatively higher mass concentration appeared in winter than that in summer. The relatively low concentration of PM_2.5_ and PM_10_ in summer related to the increase of strong convective air mass, precipitation and larger boundary layer height in summer. In the summer of 2017, the monthly precipitation was 117.7 mm, and the precipitation days accounted for about 40.2%.

In addition, four peaks of particulate matters mass concentration observed in the month of January, March, October, and December, respectively. The highest monthly average concentration of PM_2.5_ and PM_10_ mainly occurred at the western region and southern region in January especially. And the highest concentration station was Liaoshenxilu station, followed by Xinxiujie station. The reasons for the highest concentration appeared in these areas may be that the western and southern regions were industrial areas and important traffic areas. Moreover, residential heating emission was also an important factor causing the high concentration of particulate matter in winter. Cheng *et al*. pointed out that residential heating emission was also an important factor contributing to the high concentration of PM_2.5_ in Beijing, in addition to geographical, meteorological factors and regional transportation of air pollutants^37^. Chen *et al*. observed that air quality in most cities in the Jing-Jin-Ji region deteriorated significantly in winter, mainly due to the additional emissions of air pollutants caused by residential heating^28^. Yang *et al*. found that high concentrations of particulate matter in many cities in northern China due to emissions from residential heating during the cold winter months^26^. The lowest monthly average mass concentration of particulate matter found at the Senlinlu station located in the north of Shenyang in August, that was, since the station was located in the forest area, it was not easy to be affected by human activities, resulting in the low emissions of local pollution sources.

Figure 2 (c,d) were the diurnal variations of PM_2.5_ mass concentrations and PM_10_ mass concentration in four seasons. The diurnal variation trend PM_2.5_ and PM_10_ in four seasons was same. Winter and spring showed the most obvious changes, followed by autumn and summer. The change of particulate matter at night (0: 00-6:00) in summer and autumn was in steady state, the trend of particulate matter at night (0: 00-6:00) in spring and winter was decrease. In spring, autumn and winter, particulate matter mass concentrations rose from 7:00 in the morning, and the trend was bimodal state throughout the day. That is, the first peak appeared from 9:00 to 10:00 in a day, and the peak of winter morning delayed 1 to 2 hours than other three seasons. It was mainly due to morning rush hour on travel, anthropogenic emission source and relative humidity was high, atmospheric boundary layer height was low, inversion layer closing to the ground appears, pollutant diffusion conditions was relatively bad. After the peak at morning, temperature increases, relative humidity decreases, atmospheric convection movement gradually strengthens, diffusion conditions improvements, traffic pollutants reduces, particulate matter mass concentrations gradually decreases, reached to the lowest point around 17:00 in the day. Particulate matter mass concentrations gradually increased after 17:00, it was due to evening rush hour, increasing of man-made emissions, occurrence of cooking fume pollution, coupled with the industrial electricity enter the cheaper stage, industrial pollution boost, so that the particulate matter mass concentration rose and remained at a high state. In addition, the diurnal variation of particulate matter was higher at night than the day, reached to the lowest point around 17:00. The four seasons have similar trends, but varying ranges with respect to different seasons. It shown from Fig. 2 (c,d) that the variation curve of the particulate matters mass concentration presented bimodal distribution in each season, in which the absolute value was largest between the peak and the valley in spring.

### The unidirectional causality influence of factors on PM_2.5_

Here, correlation analysis and path analysis were used to quantify the unidirectional causality effect of influence factors on PM_2.5_ concentration.

**Figure 3:**
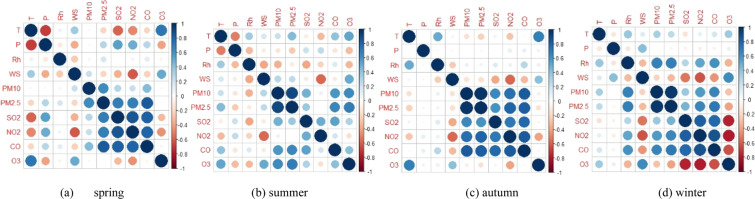
Correlation of the air pollutants and the meteorological factors. Blue meant the two variables were positive correlate, and red meant the variables were negative correlate. The darker the color, the greater the correlation of the variables.

The Pearson correlation were analyzed between six air pollution, i.e., PM_2.5_, PM_10_, SO_2_, NO_2_, CO and O_3_, and the common meteorological factors(atmospheric pressure, temperature, wind speed and relative humidity) in different seasons in Shenyang. According to the Pearson correlation coefficient shown in Fig. 3, the meteorological factors affecting different air pollutants varied in different seasons. In spring, such as PM_2.5_, SO_2_, NO_2_, CO and O_3,_ they were more susceptible to the changes of temperature, wind speed and atmospheric pressure, but the correlation between them with the relative humidity was not significant. Pearson coefficient indicates that O_3_ was positive correlated with temperature, indicating that O_3_ has strong dependence on temperature. Atmospheric pressure was positive correlated with PM_2.5_ and PM_10_, which resulted from the fact that atmospheric pressure obstructs the upward movement of particulate matter, and leaded to the accumulation of particulate matter. In summer, the relative humidity was negative correlated with PM_10_, SO_2_, NO_2_ and O_3_. This was mainly due to the low relative humidity and strong wind that easily carry surface dust, and they could combine the dust with water vapor, and easily formed fog and haze, and then made gaseous pollutants not easy to spread. SO_2_ had strong negative correlation with wind speed, CO has positive correlation with temperature and relative humidity, and NO_2_ has strong correlation with temperature, atmospheric pressure, wind speed and relative humidity. PM_10_ and O_3_ were positive correlated with wind speed, which was obviously due to strong wind speed in summer, which caused dust suspension and regional transport of O_3_. Particulate matter concentration was positive correlated with temperature, indicating that secondary particles transformed by photochemical process at higher temperature. Temperature was the main factor of O_3_, The formation of O_3_ depended on the intensity and duration of solar radiation. In autumn, wind speed was an important meteorological factor affecting most pollutants. SO_2_, NO_2_, CO, PM_10_ and PM_2.5_ were negative correlated with wind speed, indicating that wind speed has important effect on pollutant diffusion in autumn. Temperature was negative correlated with NO_2_, PM_10_ and PM_2.5_, mainly due to heating emission caused by temperature decline. In winter, relative humidity and wind speed were significant meteorological factors affecting pollutant. O_3_ was negative correlated with relative humidity, which caused by reduced visibility under high humidity conditions. Low visibility can weaken photochemical activity, thus reducing O_3_ concentration. It can be found that PM_2.5_ was significant positively correlated with relative humidity only in winter, but insignificant in other seasons. SO_2_, NO_2_ and CO are positive correlated with relative humidity and significantly negatively correlated with wind speed. PM_10_ and PM_2.5_ are positive correlated with relative humidity and negative correlated with wind speed. In almost all seasons, particulate matter concentration and four gas pollutants were negative correlated with wind speed, which indicated that strong horizontal diffusion reduce the particulate matter concentration, especially PM_2.5_. Particulate matter concentration correlated with relative humidity, which indicated the importance of aerosol particles hygroscopicity. In addition, these gas pollutants also correlated significantly with the relative humidity in autumn and winter. Whether other meteorological factors significantly affected the gaseous pollutants depended on different seasons. As shown in Fig. 3, temperature and air pressure were the main factors affecting PM_2.5_ in spring and summer, while in autumn and winter temperature was no longer the main factor affecting PM_2.5_. Instead, wind speed had significant negative correlation with PM_2.5_. The relative humidity was positive correlated with PM_2.5_ only in winter and they had no significant correlation in other seasons. Wind speed was the main factor affecting PM_10_. The relative humidity correlated with PM_10_ significantly only in summer and winter. Temperature and air pressure had no significant correlation with PM_10_. In general, wind speed was the most important meteorological factor affecting particulate matter mass concentration, and temperature, air pressure and relative humidity were also the key affecting factors in some seasons.

Considering gaseous pollutants in the atmosphere possibly formed secondary particles through atmospheric chemical reactions to realize the transformation from gas to particles. Pearson method used to discuss the effects of four gaseous pollutants and PM_10_ on PM_2.5_ (Fig. 3). It found that PM_2.5_ had significant positive correlation with PM_10_, SO_2_, NO_2_ and CO, and weakly correlates with O_3_.

In addition to the analyzing for unidirectional causality influence of factors of meteorological factors and air pollutants on PM_2.5_, path analysis used to further estimate the direct and indirect effects of meteorological factors on air pollutants in different seasons.

**Figure 4:**
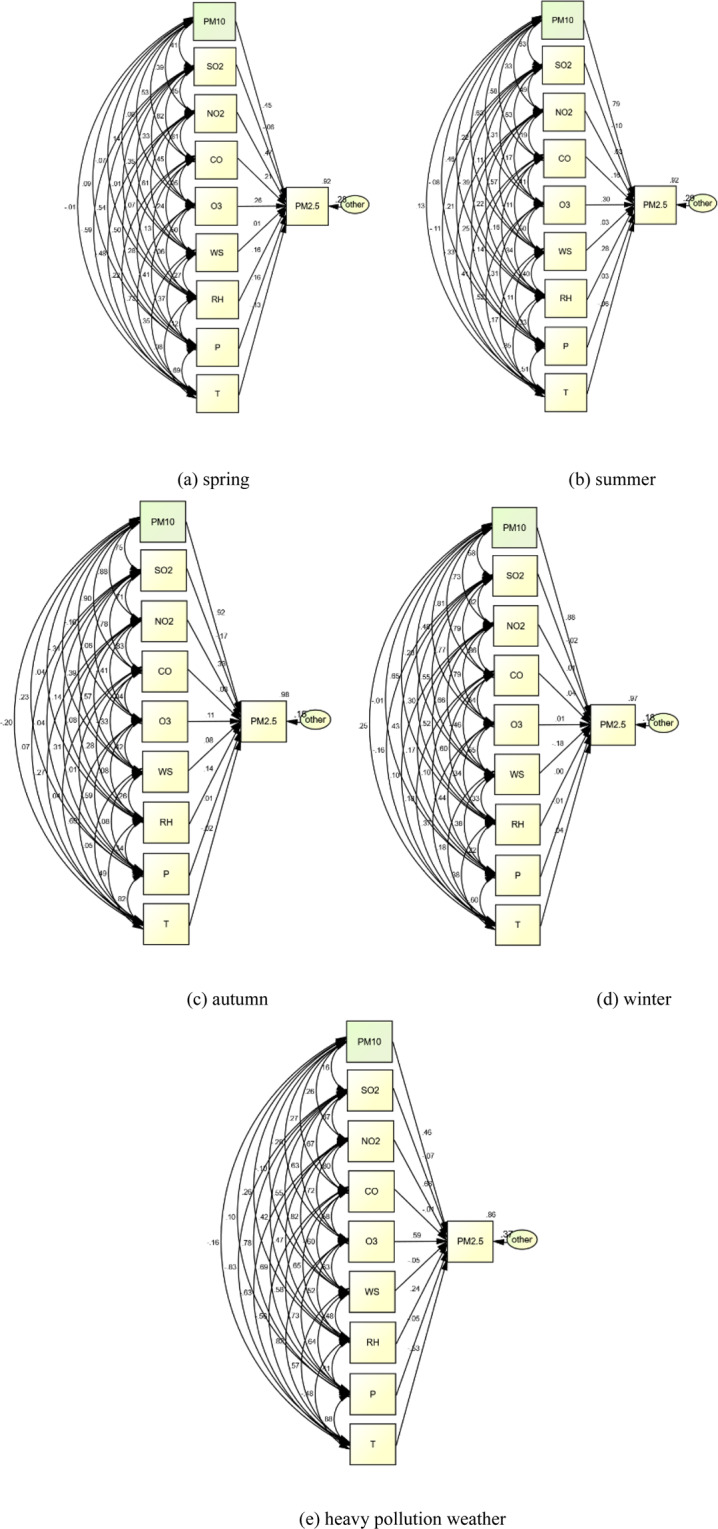
Path analysis of the air pollutants and the meteorological factors.

Path analysis results (Fig. 4) showed that: In spring, NO_2_ has the largest direct effect on PM_2.5_, while SO_2_ and CO had the largest indirect effect. CO and NO_2_ played the biggest role in direct and indirect effects, and atmospheric pressure and relative humidity played the greatest positive effect. In summer, O_3_ had the largest direct effect on PM_2.5_, followed by relative humidity. While, SO_2_ and O_3_, as well as temperature had the largest indirect effect. The pollutants with the largest combined effects of direct and indirect effects were SO_2_ and O_3_, and the meteorological factors were temperature and wind speed. In autumn, the most direct effect on particulate matter was NO_2_, followed by relative humidity. The most indirect effect was CO, SO_2_ and NO_2_. The pollutants with the largest combined effect of direct and indirect effects were CO, SO_2_ and NO_2_. In winter, CO and temperature has the largest direct effect on particulate matter, while CO, SO_2_ and NO_2_ have the largest indirect effect. The pollutants with the largest combined effect of direct and indirect effects were CO, SO_2_and NO_2_. The meteorological factor with the biggest positive effect was relative humidity, and the biggest inhibiting was wind speed.

The above analysis results indicated that NO_2_ and CO were the key factors affecting PM_2.5_ in Shenyang. This showed that the incomplete combustion of coal in autumn and winter in Shenyang increased CO concentration in the air, and PM_2.5_ concentration increased accordingly, incomplete combustion in the heating process can be considered more. Secondly, NO_2_ was another major factor affecting PM_2.5_ concentration in multiple seasons, which indicated that vehicle exhaust and industrial exhaust gas contributed to the increase of PM_2.5_ mass concentration.

Finally, path analysis was used to consider the heavy pollution weather throughout the year(Fig. 4), that was, PM_2.5_ and PM_10_ concentration were higher than level 2 respectively (PM_2.5_ > 75, PM_10_ > 150). The results showed that the positive direct action of NO_2_ and the negative direct action of temperature were the largest. The positive indirect effect of CO and SO_2_, the negative indirect effect of O_3_ and wind speed were the largest. Therefore, the positive comprehensive effect of NO_2_ and CO was the largest, and the negative comprehensive effect of wind speed and temperature was the largest. This was consistent with the results of the four seasons.

### Spatial relationship between air pollutants and meteorological factors

**Figure 5:**
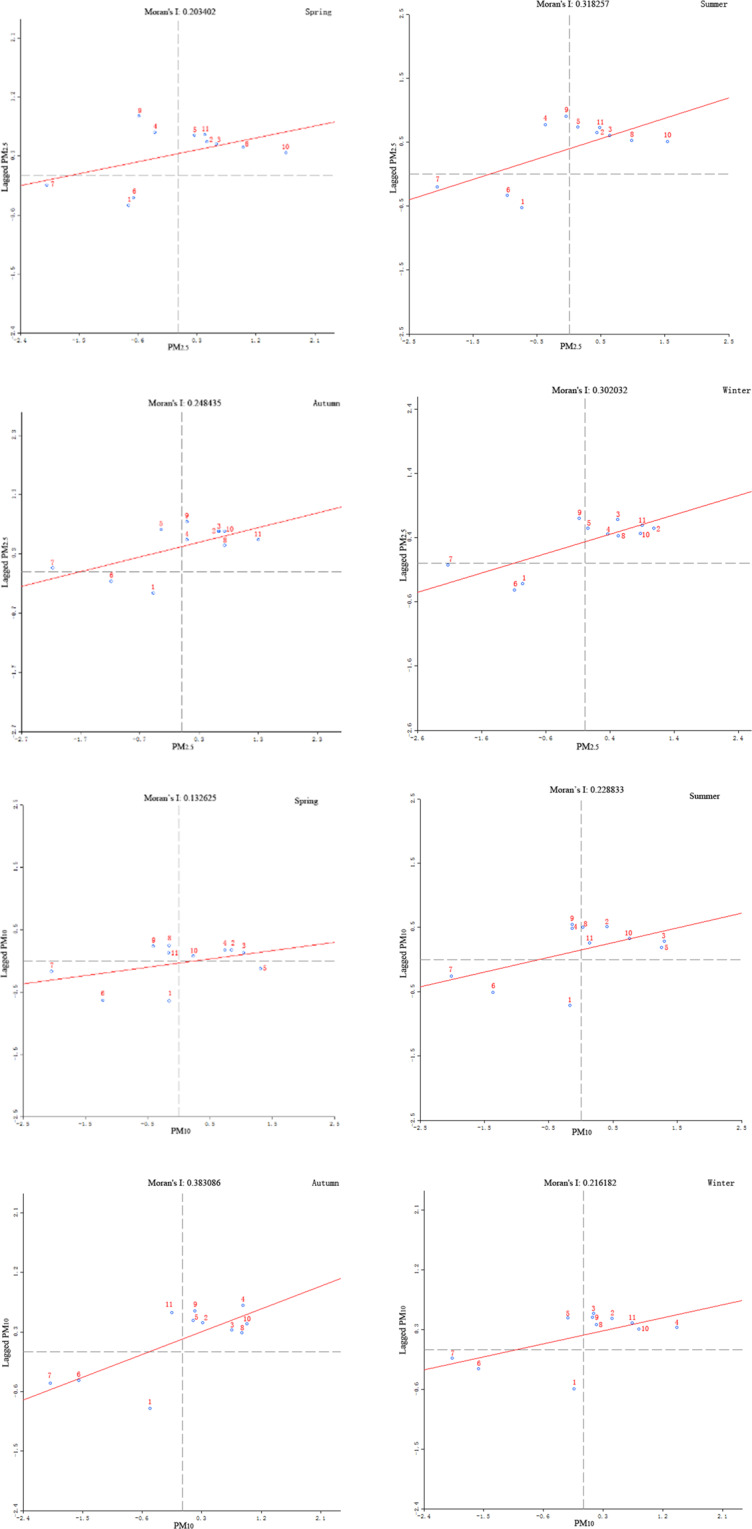
The Moran’s I value scatter of PM_2.5_ and PM_10_ in Shenyang.

Moran’s I scatter diagram obtained for particulate matter in Shenyang to test the spatial autocorrelation of PM_2.5_ and PM_10_ (Fig. 5). The station numbers showed in Table 1. The results showed that the Moran’s I index of PM_2.5_ and PM_10_ in the four seasons were greater than 0 and their p values were all less than 5%, which meant that both PM_2.5_ and PM_10_ showed significant spatial autocorrelation among the different monitoring points.

Moran’s I scatter diagram were divided into four quadrants, which corresponded to: high-high concentration area (upper right), low-low concentration area (lower left), low-high concentration area (upper left) and high-low concentration area (lower right). The high-high concentration area was also known as hot spot, indicating that these areas were at the high risk level; The low-low correlation concentration area was cold spot, showed low risk level. The low-high concentration area showed that low concentration zone was surrounded by high concentration zone. The high-low correlation area was just the reverse with the low-high correlation area.

As could be seen from Fig. 5, most of the monitoring stations such as Xiaoheyan, Wenhualu, Lindongjie, Hunnandonglu, Xinxiujie, Jinshenjie and Liaoshenxilu were located in the high-high concentrated areas, i.e. hot spot, there PM_2.5_ mass concentration was higher than other areas in Shenyang city. These stations were mainly located in the center and the southwest of Shenyang city and showed strong spatial correlation. The high-high concentration area remained unchanged with the change of seasons. The stations of Donglinglu, Senlinlu and Yunonglu that were located in the eastern and northern of Shenyang city belonged to the low-low concentration area, namely cold spot area, where PM_2.5_ concentration was low. The low-low area varied little with the seasons. In addition, although Taiyuanjie monitoring station was also located in the central of Shenyang, it belonged to the low-high area, which surrounded by high pollution area. The low-high region remained unchanged in spring and summer, but moved northward in autumn.

The mass concentration distributions of PM_10_ were slightly different from that of PM_2.5_ (Fig. 5). In terms of PM_10_, the stations distribution in high-high concentration area was only one less than that of PM_2.5_, which is Xiaoheyan station. The high-high aggregation area in spring and autumn was more widely distributed. The low-low aggregation area of PM_10_ were the same stations with that of PM_2.5_. Both Xiaoheyan and Taiyuan streets were in the low-high area surrounded by high pollution.

The number monitoring sites distributed in the high-high concentration areas was far more than that distributed in low-low concentration areas for both the PM_10_ and PM_2.5_. It indicated that the number of high-value concentration areas was far more than that of low-value concentration areas, and the distribution range was wide. This suggests that the joint prevention and control between regions was a necessary measure to reduce particulate matter concentration. The aggregation areas of PM_10_ and PM_2.5_ had little changes throughout the year. It meant that the spatial correlation analysis results of urban agglomeration in winter and summer were consistent. It indicated that apart from meteorological factors and heating, industrial structure layout of energy consumption between these areas were also an important factor affecting particulate matter concentration. Therefore, planning the industrial layout and adjusting the industrial structure were one of the important means to reduce particulate matter concentration.

### High particulate matter mass concentration analysis

**Figure 6:**
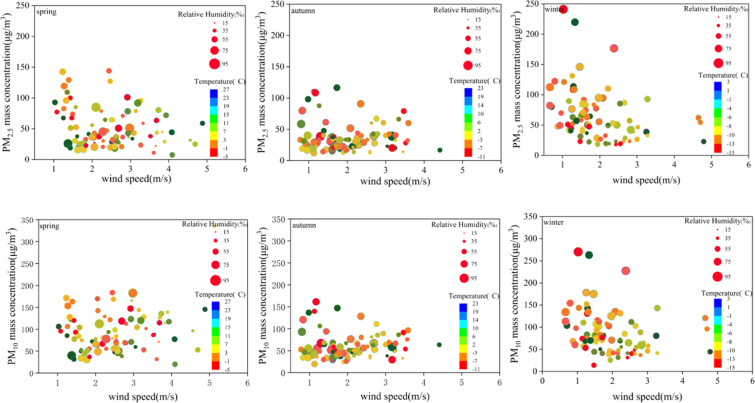
Average daily level of particulate matter under different conditions of temperature, relative humidity and wind speed. The circle size meant relative humidity. The color meant temperature values.

According to the previous analysis in this research, particulate matters concentration closely related to meteorological conditions. Heavy pollution occurred most often in winter, spring and autumn. As shown in Fig. 6, conditions with low wind speed, low temperature and low relative humidity in spring, with low temperature and high relative humidity in autumn, and with low wind speed, low temperature and high relative humidity in winter, pollution weather with particulate matters reaching level 2(fair level) or above was likely to occur. Regional emergency mitigation measures should be taken earlier to prevent and control heavy pollution weather under adverse meteorological conditions.

**Figure 7:**
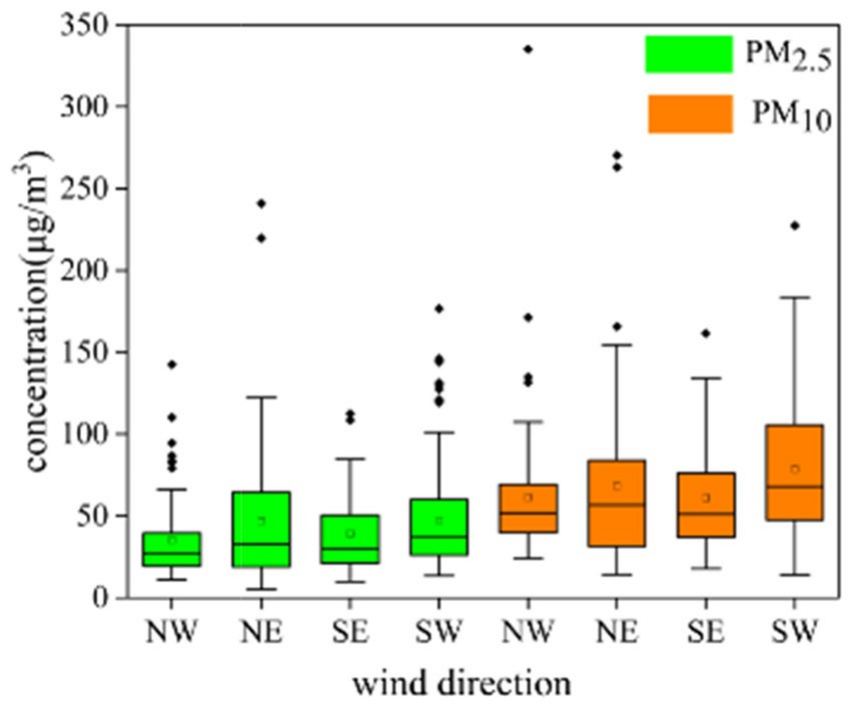
Box-Whiskers plot of particulate matter mass concentration related with four different kinds of wind direction. The bottom and top of each box meant the 25th and 75th percentiles, and the vertical dotted lines at the bottom and top meant the minimum and maximum values. The black solid line in the box meant median value. Square meant the average value. “◆” denoted outlier. NW meant northwest wind. NE meant northeast wind. SE meant southeast wind. SW meant southwest wind.

The particulate matter mass concentration values with different wind direction shown in Fig. 7. It found that high particulate matter mass concentration mainly accompanied by southwest wind, while low particulate matter mass concentration occurred at the same time as northwest wind. As PM_10_ was heavier than PM_2.5_, it was not easy to transport in a long distance, so the problem of local pollution in the southwest was more serious.

## Conclusions

In this paper, six kinds of air pollutants and five meteorological factors collected in Shenyang during 2017 based on the day observations at 11 monitoring stations. The influencing factors of particulate matter analyzed. Several main conclusions shown as follows:

1. The annual temporal variation of PM_2.5_ and PM_10_ presented a saddle-like distribution pattern along with the observation days. The monthly average change pattern of PM_2.5_ and PM_10_ showed similar trends and the mass concentration in winter was higher than that in summer. The diurnal variation trend was bimodal state throughout the day and in winter and spring the diurnal variation was obvious, followed by autumn and summer.
2. Particulate matter concentration closely related to meteorological conditions. Meteorological factors affecting air pollution most were different in each season in Shenyang. Wind speed was the most important meteorological factor affecting PM_2.5_ and PM_10_, temperature, air pressure and relative humidity also the key affecting factors in some seasons. In addition to the meteorological factors, the possible air pollutants affecting particulate matter considered, and the analysis revealed that PM_10_, NO_2_ and CO were also the key factors affecting PM_2.5_ in Shenyang. The adverse meteorological conditions tended to form severe pollution weather with high particulate matters concentration in some seasons. High particulate matter mass concentration mainly accompanied by the southwest wind, while the low particulate matter mass concentration occurred with the northwest wind.
3. PM_2.5_ and PM_10_ showed significant spatial autocorrelation. The changes in the aggregation area of PM_2.5_ and PM_10_ in each season was small. The number of high-value aggregation areas was much higher than that of low-value aggregation areas and the variation range was wide, which indicated that regional joint prevention and control measures should strengthened.

## Methods

There were 11 air quality monitoring stations in Shenyang, covering eastern area, southern area, western area, northern area and central area (Table 1). Air pollutants for 11 monitoring stations at Shenyang in 2017 were used in this study, including PM_2.5_, PM_10_, SO_2_, NO_2_, CO and O_3_ collected from China air quality monitoring platform. Meteorological data obtained from Climate daily data sets in ground international exchange station of China meteorological administration, including atmospheric pressure, wind speed, wind direction, temperature and relative humidity. The average daily data of particulate matters mass concentration and meteorological factors collected during 1 January 2017 to 25 December 2017. The daily average concentration of SO_2_, NO_2_, CO and O_3_ collected during 1 January 2017 to 30 October 2017.

The descriptive statistics and trend plots on particulate matter mass concentration were conducted by Origin2017(1991-2016 OriginLab Corporatin, USA). The maps in Fig. 1 conduced based on ArcGIS 10.5(1999-2016 Esri Inc, 10.5.0.6491). The correlation between particulate matter mass concentration, meteorological factors and other air pollutants analyzed using the method of Spearman correlation. Spearman correlation analysis was calculated by IBM SPSS Statistics 22.0.0.0 (IBM Corporation and other(s)1989, USA).The calculation formula of Spearman correlation coefficient was as follows:

$$\rho =\frac{{\sum }_{\text{i}}({x}_{i}-\overline{x})({y}_{i}-\overline{y})}{\sqrt{{\sum }_{\text{i}}{({x}_{i}-\overline{x})}^{2}{({y}_{i}-\overline{y})}^{2}}}$$

Where $x$ is daily average concentration of air pollutants, and $y$ is the daily average concentration of meteorological factors. When $y$ tends to increase with the increase of $x$, the spearman correlation coefficient is positive. Conversely, Spearman correlation coefficient was negative.

Path analyses used to evaluate the direct and indirect path coefficients that could explain the effects of meteorological factors, NO_2_, SO_2_, CO and O_3_ on particulate matter. Path analysis conducted by IBM SPSS Amos 22.0.0(IBM Corporation and its licensors 1983.2013, USA). The general method and steps of path analysis were as follows:

1. The canonical equation of normalized linear regression is:
  $${R}_{\text{xx}}{b}^{\ast }={R}_{\text{xy}},$$
  ${R}_{\text{xx}}$ is the correlation matrix for ${x}_{1},{x}_{2},\cdots ,{x}_{p}$, ${b}_{j}^{\ast }={({b}_{1}^{\ast },{b}_{2}^{\ast },\cdots ,{b}_{p}^{\ast })}^{T}$, ${b}_{j}^{\ast }$ is the direct effect of ${x}_{j}$ on $y$, ${r}_{jk}{b}_{k}^{\ast }$ is the indirect influence of ${x}_{j}$ on $y$ through ${x}_{k}$, ${R}_{\text{xy}}$ is the correlation matrix of $x$ to $y$;
2. According to the equation ${R}_{\text{xx}}{b}^{\ast }={R}_{\text{xy}},$ get the path coefficient ${b}_{j}^{\ast }={R}_{\text{xx}}^{-1}{R}_{\text{xx}}$, Where ${R}_{\text{xx}}^{-1}$ is the inverse matrix of ${R}_{\text{xx}}$;
3. The decision coefficient can be obtained from the path coefficient:
  $$\{\begin{array}{c}{\text{R}}_{j}^{2}={b}_{j}^{\ast 2}\\ {R}_{\text{jk}}={R}_{\text{kj}}=2{b}_{j}{r}_{jk}{b}_{k}\end{array}$$
  Where ${\text{R}}_{j}^{2}$ is direct determination coefficient of ${x}_{j}$ to $y$, and ${R}_{\text{jk}}$ is the indirect determination coefficient of ${x}_{j}$ about $y$ through ${x}_{k}$.
4. The total decision coefficient of ${x}_{j}$ about $y$ is $R(j)={R}_{j}^{2}+{\sum }_{j\ne k}{R}_{\text{jk}}=2{b}_{j}^{2}{r}_{jk}-{({b}_{j}^{\ast })}^{2}$, $j=1,2,\cdots ,p$.

The spatial autocorrelation method used to measure the spatial autocorrelation of particulate matter. It conduced based on GeoDa1.2.0 (2011, 2012 by Luc Anselin, Chicago, USA). Spatial autocorrelation usually tested by Moran’s I index. The calculation formula of Moran’s I index got as follows:

$$I=\frac{({x}_{i}-\bar{x})}{{S}^{2}}\sum _{j}{w}_{ij}({x}_{j}-\bar{x}),\,{S}^{2}=\frac{1}{n}{\sum _{j}({x}_{i}-\bar{x})}^{2},\,\bar{x}=\frac{1}{n}\mathop{\sum }\limits_{i=1}^{n}{x}_{i},\,w=[\begin{array}{cccc}{w}_{11} & {w}_{12} & \cdots  & {w}_{1j}\\ {w}_{21} & {w}_{22} & \cdots  & {w}_{2j}\\ \cdots  & \cdots  & \cdots  & \cdots \\ {w}_{i1} & {w}_{i2} & \cdots  & {w}_{ij}\end{array}]$$

Where $n$ is the number of monitoring points. ${w}_{ij}$ is the value of the space weight matrix $w$, which is equal to 1 when the area of monitoring point $i$ is adjacent to the area of monitoring point $j$, otherwise it is equal to 0. ${x}_{i}$, ${x}_{j}$ respectively represent the pollutant concentration value of monitoring point $i$ and $j$. The value of Moran’s I index is generally between $[-1,1]$. When $I > 0$, it means that it is a positive spatial correlation, and the values clustered high in space. When $I < 0$, it means that it is a negative spatial correlation, and the values clustered high-low in space; When $I=0$, it means that there is no spatial correlation. P value is the significance of Moran’s I index. When P is lower than 0.05, the spatial correlation is considered significant; otherwise, it is not significant.
